# Book Reviews

**Published:** 1895-02

**Authors:** 


### *BOOK REVIEWS.*

---

The Principles of Surgery and Surgical Pathology. General Rules Governing Operations and the Application of Dressings. By Dr. Hermann Tillmanns, Professor in the University of Leipzig. Translated from the third German edition by John Rogers, M.D., and Benjamin Tilton, M.D., New York. Edited by Lewis A. Stimson, M.D., Professor of Surgery in the University of the City of New York, Medical Department. With 441 Illustrations. D. Appleton & Co., New York, 1894.

This is the first time that this work has appeared in this country, but it has enjoyed a wide circulation in Europe.

It is divided into three sections. Section first gives the technique of operative surgery, the preparations for aseptic operations, etc. Asepsis is advocated instead of antisepsis, and it is proven that wounds heal more readily with the aseptic treatment. In this section anesthesia is discussed. On page 15 some vile typographical errors occur in the names of Dr. W. T. G. Morton and Dr. C. W. Long. Dr. Long is given precedence in the use of ether, though under a name which even his friends could hardly recognize. A great disproportion exists in the space given to chloroform and ether, to the disadvantage of the latter. No mention is made of the *A. C. E.* mixture. Various methods are described for preventing the loss of blood during an operation, but nothing said about Wyeth's pins. The next section is an excellent description of the principles governing antiseptic and aseptic dressings, the choice of antiseptics, etc. The author advocates the dry dressing of wounds; and he never uses dusting powders (iodoform, boric acid, etc.) to wounds which have been sutured. Infected wounds, of course, call for antiseptic treatment. Section third embraces the greater part of the work, and describes in detail the phenomena of inflammations and injuries, and the diseases due to systemic infection, such as erysipelas, tetanus, septicemia and pyemia, tuberculosis, syphilis, etc.; injuries and surgical diseases of the soft parts, bones, and joints are also taken up; and the section closes with a complete description and classification of tumors. Bacteriology in its relation with pathogenesis of diseases receives proper consideration. This part of the work is especially well illustrated.

This is an excellent work, and cannot fail to commend itself highly to those who wish to know more of surgical pathology than can be found in the usual text-books on general surgery. Its teachings represent, better than any other book that we know of, the principles which underlie modern surgical practice.

---

OBSTETRIC SURGERY. BY Egbert H. Grandin, M.D., Obstetric Surgeon to the New York Maternity Hospital, Gynecologist to the French Hospital, etc.; and George W. Jarman, M.D., Obstetric Surgeon to the New York Maternity Hospital, Gynecologist to the Cancer Hospital, etc. With eighty-five Illustrations in the Text and fifteen full-page Photographic Plates. Extra cloth, $2.50 net. Philadelphia: The F. A. Davis Co., Publishers, 1914 and 1916 Cherry street.

This book will supply one of the few remaining long-felt wants. It deals in a concise and practical manner with those surgical procedures which are sometimes required of the practitioner in his obstetric work; such as the induction of premature labor, the use of forceps, Cæsarean section, symphyseotomy, embryotomy, repair of the perineum, etc. The style is very clear, and the teachings represent accurately the latest principles and practice of the obstetrical art. Some of the author's directions about securing thorough antisepsis (by chemical agents) we believe to be almost impossible to carry out in general practice. Chapter V. describes the indication and technique of symphyseotomy. The authors think this operation is now well established. We are glad to see that they advocate the performance of symphyseotomy, or Cæsarean section, in the place of embryotomy of the living fetus in cases where election is possible. The practitioner will find this work very helpful indeed; much more so than the usual text-books on obstetrics.

ANNUAL OF UNIVERSAL MEDICAL SCIENCES. Edited by Charles E. Sajous, M.D., together with over seventy associate editors. In five volumes. F. A. Davis Company, Philadelphia, Penn., 1894.

Sajous' Annual, as this work is familiarly known, has been before the public since the issue of 1888, and has improved with each succeeding issue. The popularity of the Annual has also increased until now there is really no library which can be complete without it, and no work of elaborate conception that does not use the Annual for reference to the current literature of any subject.

"In union there is strength," and "in the multitude of council" there is wisdom, or "safety," to be more exact. Here we have not the views of any one man on a subject, but the results of the experience of thousands on one subject. The literature is so condensed, too, that the busy practitioner can find in ten lines what was originally written in ten pages, perhaps, and he never sees at all the unimportant matters. Provided he has the time and the journals at hand, he can find references at every point as to the subject in detail, and can in this way go to the bottom of the whole matter. The articles themselves are again sifted down by the index, which is so divided that it is possible to find the therapeutical suggestions that have been made for each disease, and in this way the progress can be seen.

The amount of material which is collected and carefully culled is truly phenomenal, including thousands of journals and *brochures* in the French, German, Italian, Spanish, English, and other languages. Clippings are made in Paris by the editorial department, with an office at No. 20 Rue de Madrid, and are mailed at intervals to each associate editor. The articles when prepared are revised by the editor in Paris. The illustrations are very full and complete. The type is large and leaded. The divisions and subdivisions of each subject are well marked; and, in fine, the whole work shows the master hand and head of the editor.

TEXT-BOOK OF HYGIENE: A COMPREHENSIVE TREATISE ON THE PRINCIPLES AND PRACTICE OF PREVENTIVE MEDICINE FROM AN AMERICAN STANDPOINT. By George H. Rohé, M.D., Professor of Therapeutics, Hygiene, and Mental Diseases in the College of Physicians and Surgeons, Baltimore; Superintendent of the Maryland Hospital for the Insane; Member of the American Public Health Association. Third edition, thoroughly revised and largely rewritten. Cloth, $3.00 net. Philadelphia: The F. A. Davis Co., Publishers, 1914 and 1916 Cherry street.

The author of this popular book on Hygiene has taken advantage of the demand for a new edition to subject his work to a thorough revision, and to make such additions as seemed advisable from the late advances in sanitary science. The present regulations of quarantine practice are fully described by Surgeon-General Walter Wyman and Dr. Geddings, of the Marine Hospital Service. Dr. A. L. Gihon, United States Navy, has revised the chapter on Marine Hygiene. A section has been added (by Professor Egbert, Philadelphia) on the methods of examining water, air, and foods. The chapters on the Removal of Sewage, the Construction of Houses, School Hygiene, and Disinfectants and Deodorants contain a great deal of excellent practical information. We think this book meets every demand for a practical text-book on modern hygiene and sanitation.

---

A COMPEND OF THE PRACTICE OF MEDICINE. By Daniel M. Hughes, M.D., Resident Physician Philadelphia Hospital; late Demonstrator of Clinical Medicine in Jefferson Medical College, etc. Fifth edition, revised and enlarged. Price, $2.50. P. Blakiston, Son & Co., 1012 Walnut street, Philadelphia.

We have several times expressed our disapproval of "compends." But the book before us proves that they can be made a success. The author has certainly solved the problem of presenting a compend, as he has modestly called it, which contains the most important facts of "practice" in compact and attractive form. It is the best compend that we ever saw. Indeed, it is more than that: it is a condensed and practical work on Practice which describes the usual diseases in a manner that is highly satisfactory, both from a literary and scientific point of view. This edition includes a section on skin diseases, which is very good, no doubt, but out of place. This work was originally designed for students, but we are told that it has been used by practitioners also. We cordially recommend it to both as an ideal compend. It is handsomely bound in morocco, with gilt edges.

---

BLOOD SERUM THERAPY AND ANTITOXINS. By George Krieger, M.D., Chicago. Published by E. H. Colegrove & Company, Chicago.

This little volume discusses first the history of blood serum therapy, and the methods which have been employed in immunizing animals and individuals against certain diseases. The second chapter discusses the nature of toxins and toxalbumins, with their modes of preparation. The greater part of the volume relates to the application of these principles to the treatment of tetanus and diphtheria. The book is a concise presentation of the general principles of serum therapy, and any one will find it both interesting and instructive.

---

LIPPINCOTT'S MAGAZINE FOR FEBRUARY, 1895.

The complete novel in the February issue of *Lippincott's* is "The Chapel of Ease," by Harriet Riddle Davis. It is a pleasant, peaceful story of rural life in Maryland, and of a young widow's somewhat complicated love-affair.

Francis Lynde, in "Quong Lee," shows that there are some good Chinamen. "A Precedent," by Alice M. Whitlock, narrates an unusual incident in a home for aged clergymen and widows of clergymen. In "An Idyl of the Forties," Champion Bissell points the consoling moral that men should marry the daughters of their first loves.

"The Fate of the Farmer," by Fred Perry Powers, is an instructive essay on the growing evils of agricultural tenancy. David Bruce Fitzgerald, in a brief and readable article, tells all that most people need to know about "The Diamond-Back Terrapin." Mrs. Caroline Earle White describes the festival of "Corpus Christi in

Seville," and Dr. Charles C. Abbott shows what one who has eyes and a love for nature may see during "A Walk in Winter."

Under the heading, "Lingo in Literature," William Cecil Elam, a Virginian, exposes the blunders made by many writers, even those of repute, in trying to reproduce negro dialect. He speaks by the card and with authority on a topic which (in fiction) is usually handled in a happy-go-lucky, hit-or-miss, guess-it-will-come-out-right manner.

Annie Steger Winston discusses "The Pleasures of Bad Taste" with much acumen. "The Beginnings of a Cavalry Troop," by Kenneth Brown, is an amusing *jeu d' esprit.*

The poetry of the number is by Florence Earle Coates, Carrie Blake Morgan, Edith M. Thomas, and Richard Stillman Powell. The latter pays a deserved compliment to Mr. Stanley Weyman's novels.

---

### THE COSMOPOLITAN.

General Lord Wolseley makes a most important contribution to the literature of the China-Japan war. In an article for the February *Cosmopolitan,* he discusses the situation and does not mince matters in saying what China must do in this emergency. Two other noted foreign authors contribute interesting articles to this number. Rosita Mauri, the famous Parisian danseuse, gives the history of the ballet, and Émile Ollivier tells the story of the fall of Louis Philippe. From every part of the world, drawings and photographs have been obtained of the instruments used to torture poor humanity, and appear as illustrations for a clever article, by Julian Hawthorne, entitled, "Salvation via the Rack." Mrs. Reginald de Koven, Anatole France, W. Clark Russell, Albion W. Tourgée, and William Dean Howells are among the story tellers for the February number of The *Cosmopolitan.*

